# Bacterial Quorum Sensing and Microbial Community Interactions

**DOI:** 10.1128/mBio.02331-17

**Published:** 2018-05-22

**Authors:** Rhea G. Abisado, Saida Benomar, Jennifer R. Klaus, Ajai A. Dandekar, Josephine R. Chandler

**Affiliations:** a Department of Molecular Biosciences, University of Kansas, Lawrence, Kansas, USA; b Department of Microbiology, University of Washington, Seattle, Washington, USA; University of Texas Health Science Center at Houston

**Keywords:** quorum sensing, antibiotics, coculture, competition, cooperation

## Abstract

Many bacteria use a cell-cell communication system called quorum sensing to coordinate population density-dependent changes in behavior. Quorum sensing involves production of and response to diffusible or secreted signals, which can vary substantially across different types of bacteria. In many species, quorum sensing modulates virulence functions and is important for pathogenesis. Over the past half-century, there has been a significant accumulation of knowledge of the molecular mechanisms, signal structures, gene regulons, and behavioral responses associated with quorum-sensing systems in diverse bacteria. More recent studies have focused on understanding quorum sensing in the context of bacterial sociality. Studies of the role of quorum sensing in cooperative and competitive microbial interactions have revealed how quorum sensing coordinates interactions both within a species and between species. Such studies of quorum sensing as a social behavior have relied on the development of “synthetic ecological” models that use nonclonal bacterial populations. In this review, we discuss some of these models and recent advances in understanding how microbes might interact with one another using quorum sensing. The knowledge gained from these lines of investigation has the potential to guide studies of microbial sociality in natural settings and the design of new medicines and therapies to treat bacterial infections.

## INTRODUCTION

Studies over the past half-century have revealed that bacteria can communicate among themselves to carry out a wide range of complex social behaviors, including cooperation. Such social behaviors are widespread in bacteria. It is now clear that social behaviors have important consequences in shaping the behavior and structure of polymicrobial communities. The developing interest in understanding bacterial social behaviors has led to innovative approaches for studying dynamic, mixed microbial communities. In particular, experiments using multiple-strain and multiple-species laboratory and infection models have provided critical new insights into bacterial sociality. In this minireview, we will focus on a type of cell-cell signaling in bacteria called quorum sensing (QS), which has emerged as one model for understanding bacterial sociality. We will review the basic molecular mechanisms of quorum sensing, primarily focusing on *Proteobacteria*. We highlight recent studies of quorum sensing that use laboratory, *in situ*, and *in vivo* models of multiple-strain and multiple-species communities and describe how these studies have contributed to our current practical and fundamental understanding of quorum sensing, communication, and competition in bacteria.

## OVERVIEW OF QUORUM SENSING

**FIG 1:**
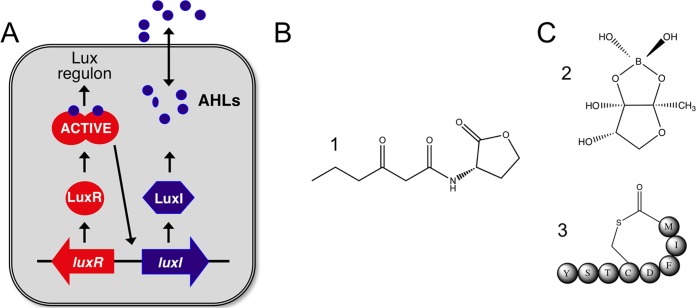
(A) AHL quorum sensing in Vibrio fischeri. AHL signals (AHLs [solid blue circles]) are synthesized by LuxI family signal synthases and specifically interact with LuxR family transcription factors. When the population reaches high cell density, accumulated AHLs interact with LuxR homologues. AHL interaction causes the LuxR protein to change conformation and become active, which induces target gene regulation. In V. fisheri, LuxI and LuxR produce and respond to, respectively, the AHL *N*-3-oxo-acyl-homoserine lactone (3OC6-HSL). (B) Structure 1, the Vibrio fischeri AHL, 3OC6-HSL. AHLs can vary in the side chain length and substitution at the third carbon position of the acyl chain, and this variation dictates the specificity of the system. (C) Structure 2, Vibrio harveyi and Vibrio cholerae AI-2, furanosyl borate ester form. Structure 3, Staphylococcus aureus autoinducing peptide (AIP-1). The letters in the balls indicate amino acids that are cyclized posttranslationally.

Quorum sensing (QS) is a type of population density-dependent cell-cell signaling that triggers changes in behavior when the population reaches a critical density (1, 2). QS systems rely on the production and sensing of extracellular signals. Typically, bacteria continually generate the signal starting at a low concentration in a fresh culture, and the signal accumulates in the local environment as the population density increases. Once a threshold concentration is reached, the signal interacts with a receptor protein, causing a coordinated change in gene expression in the population. Several types of QS signals exist: many *Proteobacteria* utilize acyl-homoserine lactone (AHL)-type signals, and Gram-positive species utilize short oligopeptide signals that are often chemically modified (Fig. 1). The QS systems in many bacterial species are well understood at the molecular level, and extensively reviewed elsewhere (2–5). In this review, we focus on models that have been developed to study how QS systems increase the success of individuals in multiple-strain and multiple-species communities. In many cases, AHL signaling systems are the focus of these studies; however, other types of QS systems have also been studied.

### AHL QS and diversity of QS systems.

AHL QS was first described in the 1960s and 1970s in the bioluminescent marine bacterium Vibrio fischeri through the identification of “autoinducing” activity in conditioned medium of high-density cultures, which controls bioluminescence as part of symbiotic associations between V. fischeri and the pinecone fish, Monocentris japonica, and the Hawaiian bobtail squid, Euprymna scolopes (for reviews, see references 6 and 7). Bioluminescence is thought to help the pinecone fish and the squid mask their shadows during predator avoidance (8). At high cell densities, V. fischeri activates bioluminescence through the QS proteins LuxR and LuxI. LuxI is a signal synthase that produces *N*-3-oxo-hexanoyl-homoserine lactone (3OC6-HSL) (9). 3OC6-HSL specifically binds to LuxR (10, 11), a transcription factor that activates expression of the *luxCDABEG* operon, which contains the genes that enable bioluminescence (12, 13) and about 20 others (14). Similar AHL QS systems have since been shown to be widely distributed in *Proteobacteria*, where they control diverse behaviors, such as production of secreted toxins and virulence factors, biofilm matrix components, and DNA conjugation (for reviews, see references 1 to 3 and 15). Many QS architectures involve more than one signal-receptor combination: for example, Pseudomonas aeruginosa has two complete LuxR-I-type circuits, LasR-I and RhlR-I, which function in a hierarchy, and Burkholderia thailandensis has three complete LuxR-I circuits. The selective pressures that result in multiple AHL signaling circuits are not clear, although it has been proposed that the different properties of AHL signals might provide specific benefits in different environments (16).

LuxR- and LuxI-type proteins have now been studied across many different bacterial species. Most LuxI-type AHL synthases produce AHLs from *S*-adenosylmethionine (SAM) and an acylated acyl carrier protein (ACP) from the fatty acid biosynthesis pathway (17–19). The fatty acyl side chains can vary in length and oxidation at the third carbon position, and this variation accounts for the specificity of the different systems. LuxR proteins contain a conserved N-terminal AHL-binding domain (11) and a C-terminal helix-turn-helix DNA-binding domain (20). The N terminus of LuxR interacts with AHLs, and this interaction induces homodimerization of the LuxR homologue and subsequent binding to a specific DNA sequence called the *lux* box in the target promoter (21, 22). The AHL-bound LuxR often induces transcription of its cognate *luxI* signal synthase gene, resulting in a positive-feedback loop that further increases the concentration of AHLs (13). Although most LuxR family proteins act as transcriptional activators, a few are repressors (for a review, see reference 23). Cognate LuxI and LuxR family proteins are often encoded adjacent to one another. However, some species, including P. aeruginosa and B. thailandensis, encode LuxR-type proteins with no cognate LuxI family AHL synthase; these are termed orphan LuxR receptors (24).

Other types of signal-receptor QS systems exist in both proteobacterial and Gram-positive organisms. There is an alternative form of QS signaling in Vibrio harveyi and Vibrio cholerae (for a review, see reference 3). QS in these species involves the signals CAI-1 and AI-2 and a third signal, HAI-1, in V. harveyi, which each specifically bind to different cell surface receptors. The receptors act in parallel to control expression of QS genes by modulating the phosphorylation state of two phosphorelay response regulators (LuxU and LuxO) (25, 26), regulatory small RNAs, and finally, a master QS gene transcription regulator (27, 28). Gram-positive bacteria have peptide-based QS systems, of which the most well studied is the *agr* system of Staphylococcus aureus (for a review, see reference 29). In this system, the QS signal is produced by AgrB and AgrD. *agrD* encodes a prosignal (30), which is exported and modified by the protein AgrB (31). At sufficient concentrations, the signal binds to the cell surface receptor AgrC, a histidine kinase that in turn activates the response regulator AgrA and affects changes in gene expression through a small RNA called RNAIII (32).

In addition to bacterial QS systems, QS-like systems have also been reported in eukaryotic microbes (33, 34), viruses (35), and even higher-order species such as ants (36, 37). These examples provide evidence that population density-dependent responses have an important role across many different domains of life. In bacteria, many of the factors controlled by QS systems are secreted or excreted goods that can be shared by the entire group and are important for cooperation (4). Some of the cooperative activities, such as secretion of toxins, might also be important for competition with other strains or species of bacteria. Studies of QS in natural mixed microbial communities are difficult because of the variability and complexity of these communities. A significant advance in QS research has been the use of models where nonclonal bacterial populations are grown in well-controlled environments. These models have provided significant new insights into the mechanisms driving microbial community interactions and how these interactions influence QS evolution. In the remainder of this minireview, we discuss the ways by which these models have shaped our current understanding of how QS control of certain factors increases reproductive success in environments where there is fierce competition for resources. Together, these studies have established a framework to think about the selective advantages and disadvantages of using QS to regulate the production of a variety of extracellular products.

## QS AND COOPERATION

### QS control of cooperative behaviors.

Many QS-regulated products are shared “public goods” that can be used by any member of the community (38). Typically these are secreted or excreted products, such as secreted proteases (39, 40). Synthesis of public goods imparts a metabolic cost for an individual cell, but is beneficial for all the other cells within the population (38). The cost involved in the production of QS-dependent public goods makes it prone to exploitation, or social cheating. Social cheating can offer growth or survival advantages to individual bacteria (41–44). Since cheaters thrive at the expense of cooperators, their presence may destabilize cooperation: if the proportion of social cheaters becomes too high, the population will no longer produce sufficient public goods. If public goods are needed for growth, the entire population stops growing and, ultimately, collapses (38, 44–46). Although cheating has typically been described as a within-species interaction, rivalrous species can also exploit the cooperative behaviors of microbes (47). Therefore, understanding cooperation and cheating within species has served as a foundation for expanding our knowledge of community interactions.

Many laboratory studies have demonstrated that QS systems can be cooperative (48–53) (Table 1). In situations where QS controls the production of public goods, the social cheaters that arise are QS receptor mutants (49). These studies rely on laboratory models of nonclonal populations grown under conditions requiring production of QS-controlled goods, such as proteases that are needed for bacteria to obtain nutrients from protein. The studies have shown that (i) cooperators have a growth advantage over cheaters when they are grown separately under these conditions, and (ii) cheaters exploit the cooperators and proliferate when they are grown as a mixed culture. The laboratory models used for these experiments have provided a critical step forward for studies to understand bacterial cooperation and polymicrobial community interactions in more natural environments.

**TABLE 1:** Models for studying QS in cooperative and competitive microbial interactions

Model reference	Species	Function
Cooperation models		
Casein liquid culture	P. aeruginosa	Cooperative protease production (46, 48, 49)
	V. cholerae	Cooperative protease production (52)
	C. violaceum	Cooperative protease production (45)
	V. harveyi	Cooperative protease production (51)
Swarming	B. subtilis	Cooperative rhamnolipid production (53, 58, 60)
	P. aeruginosa	Cooperative rhamnolipid production (59)
Biofilm	P. aeruginosa	Cooperative protease production (56)
*Ex vivo* infection	P. aeruginosa	Cooperative virulence factor production (64)
*In vivo* infection	P. aeruginosa	Cooperative virulence factor production (62)
	S. aureus	Cooperative virulence factor production (50)

Competition and models of multispecies interactions		
Dual-species liquid culture	B. thailandensis-C. violaceum	Antimicrobial production and AHL-dependent eavesdropping (104)
	Serratia plymuthica*-*Escherichia coli	Antimicrobial production (140, 141)
	P. aeruginosa*-*B. cepacia species	Antimicrobial production (47, 134)
	P. aeruginosa-A. tumefaciens	Antimicrobial production (133)
	P. aeruginosa-S. aureus	Antimicrobial production (47)
	P. aeruginosa-B. cenocepacia	AHL-dependent eavesdropping (113)
Biofilm	B. thailandensis	Contact-dependent toxin delivery (40, 83)
	P. aeruginosa-A. tumefaciens	Antimicrobial production and swarming (133)
	S. plymuthica-E. coli	Antimicrobial production (140)
	S. gordonii-P. gingivalis	Biofilm growth (AI-2 [142])
	S. oralis-A. naeslundii	Biofilm growth (AI-2 [143])
*In situ*	P. aureofaciens/P. fluorescens*-*Gaeumannomyces graminis	Phenazine production (102)
	P. syringae*-*plant epiphytes	Plant virulence, AHL-dependent eavesdropping (112)
Mimicking *in vivo* growth	P. aeruginosa-S. aureus	AHL-dependent competition (135)
	E. faecalis	Conjugation (136)

### Laboratory models of cooperation.

QS regulation of cooperation and cheating was first demonstrated using P. aeruginosa. P. aeruginosa uses QS to control production of a protease called elastase (54). Elastase production is required for growth when populations are grown on casein as the sole source of carbon and energy (48, 49). When P. aeruginosa populations are passaged in casein broth, mutations in the gene encoding the QS receptor LasR emerge within <100 generations. The mutations are typically single-nucleotide changes that abolish or significantly reduce LasR function (49). These mutants are cheaters, as they are unable to grow by themselves in casein broth; however, they proliferate when grown in a mixed culture with the wild type. In the passaged populations, LasR mutants rapidly increase until they reach 25 to 50% of the population. It is thought that the mutants are maintained at this frequency by a policing mechanism (55) that will be discussed below. Similar models of cooperation based on protease production have been developed using Chromobacterium violaceum (45), V. harveyi (51), and Vibrio cholerae (52). A protease-dependent laboratory model has also been used to demonstrate QS-dependent cooperation of P. aeruginosa cells grown under biofilm conditions (56).

Other systems for studying cooperation and cheating have since been developed. For example, swarming is a social trait due to cooperative production of secreted surfactants (57–59). QS controls surfactant production, and thus swarming, in several bacterial species, including P. aeruginosa and Bacillus subtilis, and has been used as the basis for a laboratory model to study QS and sociality (53, 58–60). QS exploitation by cheaters has also been demonstrated using *in vivo* models (50, 61, 62). Rumbaugh and colleagues (62) demonstrated that P. aeruginosa LasR mutants act as cheaters during wound infections in mice. In the study, LasR mutants were attenuated compared to the wild type in single-strain infections. However, when a mixture of both strains was used as an infection inoculum, the LasR mutants outgrew the wild type and ultimately dominated the population. Further, mice infected with the mixed population had reduced virulence relative to wild-type-infected mice (62). Similar results were observed with S. aureus and AgrC-null QS mutants in a wax moth larva infection model (50). Together, these experiments show that QS in many species of bacteria can be exploited under certain laboratory and infection conditions.

It is possible that QS-null bacteria have nonsocial advantages in some settings (63, 64). Surveys of some communities from infections and other environments indicate QS mutants (signal receptor deficient) can readily be isolated from diverse bacterial species, including P. aeruginosa (65–74), V. cholerae (75), S. aureus (76–78), and Enterococcus faecalis (79). However, it remains unclear whether these mutants function as cheaters in these natural communities. In one study of P. aeruginosa infections of ventilator-intubated patients, LasR mutants were shown to proliferate only when QS-intact cells were present (80), supporting the idea that the LasR mutants are social cheaters. However, in other studies, LasR mutants appear to have an intrinsic growth advantage and to be better adapted to certain growth environments than the wild type, suggesting there is a selective advantage of mutating LasR (63, 64). These types of experiments highlight the complexity of QS systems and the need for robust experimental systems in which social and nonsocial behaviors can be disentangled. Information from studies of laboratory models can reveal conditions and circumstances where social or nonsocial behavior is favored in natural communities. These systems are also useful to understand other aspects of QS and sociality, such as how QS contributes to the control of cheating.

### Mechanisms to stabilize cooperative behaviors that rely on QS.

Because the rise of cheaters can threaten cooperation in a population, a recurring question in evolutionary biology is how do cooperative systems persist, despite the ongoing threat of cheating (81)? Microbial systems are emerging as an excellent tool for studying cheater control because microbes have the advantage of rapid growth, high population yields, and reproducible growth in the laboratory. Studies in these systems suggest cheater control is widespread in bacteria (38). The laboratory models of QS and cooperation such as those highlighted in the previous section are particularly straightforward. These systems have provided novel insights into cheater control and proven a valuable tool for biologists to study cooperation and cheating.

QS can stabilize cooperation by decreasing the incentives to cheat or by sanctioning cheaters. One such mechanism of cheater control is through pleiotropy, where QS coregulates public goods with goods that provide an individual benefit (private goods) (45, 46, 58, 82, 83). A similar phenomenon occurs in the slime mold Dictyostelium discoideum in a process that does not involve QS (84). Linking public and private goods through pleiotropy causes a disincentive to cheat due to loss of the private good. In P. aeruginosa, in addition to the public good elastase, the LasR-I QS system controls a periplasmic enzyme important for adenosine catabolism, a “private good” (46). When P. aeruginosa populations are passaged on adenosine-supplemented casein medium, LasR mutant cheaters do not emerge as they do when casein is the sole carbon and energy source (46). The LasR mutants are constrained by the availability of adenosine, which provides a direct benefit to QS-proficient cooperators in the population (46). Cheater control through pleiotropy is also a feature of C. violaceum, in which QS coregulates production of a secreted protease with a membrane-localized antibiotic efflux pump (45). QS mutants are more sensitive to certain antibiotics and do not emerge when cooperating populations are passaged in the presence of these antibiotics (45). In the case of C. violaceum, QS stabilization of cooperation relies on antibiotics produced by other species. Although pleiotropic mechanisms can stabilize QS, it is thought that properties other than cheater control drive selection of QS regulation of private goods (85). In the case of adenosine catabolism and antibiotic resistance, these advantages remain unclear.

QS can also stabilize cooperation through a mechanism involving selective harm of cheaters, a type of policing or enforcement mechanism similar to that described in animals (86). Cheaters are typically punished through intoxication by factors produced by cooperators. In P. aeruginosa, QS controls production of hydrogen cyanide and also the induction of cyanide resistance. In cooperating populations grown on casein, cyanide produced by cooperators limits growth of LasR mutants (55). Interestingly, growth under certain conditions can enhance policing effects, leading to greater stability of cooperation (87). Another form of policing is observed in Burkholderia thailandensis, where QS controls a type VI secretion (T6S) toxin immunity system (83). In T6S systems, a toxin is transferred from a donor to a recipient cell during direct contact. Cells that make an immunity protein, typically close relatives (kin) of the donor, can defend against the toxin. Cells with no immunity protein are killed (88, 89), allowing kin discrimination. In B. thailandensis, QS controls both toxin delivery and toxin immunity; thus, QS-defective cheaters are sensitive to killing by cooperator-produced T6S toxins (83).

Cheating can also be deterred through “metabolic prudence”—that is, delaying production of costly products until nutrients required for growth are exhausted (57). QS provides one means of delaying production of costly public goods. For example, in swarming P. aeruginosa colonies, cheaters exploit cooperating cells that secrete rhamnolipid biosurfactants, which are needed to swarm (57). However, the cheaters swarm as well as the wild type when mixed in the same colony. This is because production of rhamnolipid is delayed until the cells are in the stationary phase, when the costs of production are relatively low. This delay was due to regulation by QS and also by nitrogen and carbon availability. The delay in producing rhamnolipids minimized the benefit of cheating, and swarming becomes cheatable when cooperators are genetically modified to produce rhamnolipids constitutively (57). Thus, QS can provide protection against cheating by delaying costly goods production.

Evolution theory predicts that limited dispersal through spatial structuring or high viscosity is also protective against cheating (90). Conditions of limited dispersal increase the probability that interacting individuals are close relatives (91), such that cooperative public goods are shared only among related cooperator cells. Indeed, QS is protected from cheaters in P. aeruginosa populations grown on casein under conditions of high relatedness (48). Many microbes grow in structured communities called biofilms, in which cell aggregates are encased in a self-produced extracellular matrix (ECM). Several recent experimental studies support the idea that biofilm formation promotes cooperation (92–95). QS controls biofilm formation in many bacterial species (for a review, see reference 96). Thus, QS-dependent biofilm formation and spatial structuring might increase cooperation, which could be important for stabilizing QS in natural environments (97).

## QS AND COMPETITION

### QS control of behaviors associated with competition.

Many bacterial species use QS to control production of secreted or cell-targeted toxins: for example, bacteriocins in *Streptococcus* species (98, 99) and type VI secretion effectors in B. thailandensis (83). (For a review of secreted QS-controlled toxins, see reference 100.) Many such toxins are thought to promote competition with other strains or species of bacteria (100, 101). Thus, QS activation would be predicted to influence species dynamics in polymicrobial communities. Early support for this idea came from studies of the wheat rhizosphere (102). In these soil communities, the saprophytes and biocontrol agents Pseudomonas fluorescens 2-79 and Pseudomonas aureofaciens 30-84 use QS-regulated antibiotic phenazines to fight the fungus Gaeumannomyces graminis var. tritici and colonize the plant. Since these early *in situ* studies, the importance of QS in competition has been demonstrated in other bacteria, primarily using laboratory models of dual-species competition (Table 1).

Why are many competition-associated factors under QS control? The QS-dependent delay in antibiotic production is thought to mitigate the metabolic costs of production until the population can produce a sufficient concentration to kill a competitor (100). This delay could also deprive competitors of the ability to mount a defensive response to subinhibitory antibiotic concentrations. Population density might also be one of several types of information used by bacteria to infer the ecologic potential for competition (101). High cell density might be a good indicator that nutrient concentrations will soon become limited and could allow regulatory changes that broadly prepare the cell for such a situation. In support of this idea, although unrelated to competition, QS regulates changes in metabolism that prepare the population for stationary-phase-induced alkaline stress (103). Although the design of studies to understand the advantages of QS regulation of competition-associated factors can be technically challenging, several models have been developed that serve as a starting point to begin to understand the role of QS in competition.

### Laboratory models of QS and interspecies competition.

Dual-species competition models present unique challenges, including differences in growth conditions and growth rates. Most model systems use species or strains with compatible growth rates and growth requirements and that are also likely to interact in natural communities. One such model uses the saprophytes B. thailandensis and C. violaceum (104). These two species grow at similar rates under the same conditions and are also both isolated from soil and water environments. In both species, QS controls the production of secreted antibiotics. In the case of B. thailandensis, the antibiotic is a ribosome-targeting polyketide, bactobolin (105, 106). The C. violaceum antibiotic active in this model is unknown. For each species, mutations disrupting QS reduce competitiveness. This dual-species competition was also used to develop an *in silico* model (104). In the *in silico* model, increasing the cost of antibiotic production or producing it too early slows population growth and decreases killing efficiency. The *in silico* results support the idea that QS regulation of antibiotic production provides a significant cost savings to populations.

The C. violaceum-B. thailandensis model also demonstrates “eavesdropping,” or detection of other species’ AHLs (104). Although many AHL receptors specifically recognize their cognate AHLs and a narrow range of structurally related analogues, the C. violaceum AHL receptor CviR has a broad spectrum of AHL response (107). In the competition model, C. violaceum CviR could detect and respond to B. thailandensis AHLs, and this ability to eavesdrop increased C. violaceum competitiveness in some situations (104). These results suggest a potential benefit of broad-range AHL detection by some LuxR homologues (108–111). This also may account for some orphan QS receptors in bacteria that lack cognate signal synthases. AHL-dependent eavesdropping has also been demonstrated for other species, such as between endophytes of plants (112) and between P. aeruginosa and Burkholderia cenocepacia, two pathogens known to coinfect the lungs of patients with the genetic disease cystic fibrosis (113). Although AHL signaling has traditionally been thought of as an intraspecies communication system, the results of these studies suggest AHLs might also be used to sense and respond to potential competitors in the environment (114–116). Another QS molecule, AI-2, is also thought to be important for interspecies signaling (117). Recent studies support that AI-2 may play a role in bacterial community dynamics in the mammalian gut (118, 119), although it is not clear if this is through a signal receptor-dependent process (120).

A substantial effort has been made to understand the interactions between P. aeruginosa and S. aureus, as these bacteria are commonly cocultured from chronic wound infections (121). P. aeruginosa and S. aureus interaction has been studied in coculture *in vitro* (122–125), in a model mimicking the wound environment (126), in an *in vivo* rat infection model (127), and using a range of clinical strains *in vitro* and *in vivo* (128) (for a review, see reference 129). When both bacteria are together *in vitro* or *in vivo*, P. aeruginosa usually surpasses or decreases the S. aureus population. This effect is largely due to compounds controlled by QS. The first such compound characterized was 4-hydroxy-2-heptylquinoline *N*-oxide (HQNO), which was originally described as an antistaphylococcal compound (130). HQNO decelerates growth by inhibiting oxidative respiration via the cytochrome system, although it is not bactericidal (130, 131). Exposure of S. aureus to HQNO does not result in eradication of S. aureus but rather in the emergence of small colony S. aureus variants (122). Another such QS-regulated antistaphylococcal compound is pyocyanin, a phenazine produced by P. aeruginosa (132). Pyocyanin, like HQNO, blocks oxidative respiration and induces the formation of small colonies (125). Finally, P. aeruginosa uses the QS-regulated protease LasA to degrade pentaglycine from the S. aureus cell wall, inducing cell lysis, which may be beneficial to P. aeruginosa (127).

The P. aeruginosa QS products hydrogen cyanide, rhamnolipid, pyocyanin, and pyoverdine are also important for interactions with *Proteobacteria* (47, 133, 134). For example, in P. aeruginosa and Burkholderia multivorans cocultures, QS-dependent production of hydrogen cyanide is important for P. aeruginosa to outcompete B. multivorans. In the experiments, QS-dependent antibiotic production could prevent B. multivorans from exploiting QS-dependent public goods (47). These results demonstrate another facet of QS-dependent pleiotropy: coregulation of antibiotics with public goods can stabilize cooperative behavior in mixed microbial communities.

### QS and models that mimic polymicrobial *in vivo* infections.

Laboratory models can be used to mimic host conditions to make inferences about the role of QS in polymicrobial infections. A recent study suggests host factors might modulate P. aeruginosa QS during coinfections with S. aureus (135). These studies were conducted using a laboratory chronic wound model that more closely mimics the chronic wound environment (135), which includes plasma and red blood cells. In the chronic wound model, plasma albumin allowed S. aureus to survive coculture with P. aeruginosa by sequestering P. aeruginosa AHLs and reducing QS activation of anti-S. aureus toxins. Because many P. aeruginosa QS-controlled toxins are also virulence factors, these results also suggest that P. aeruginosa virulence might be reduced by albumin-dependent QS inhibition during infections.

Serum can also change cell-cell interactions by modulating signaling in the Gram-positive species Enterococcus faecalis (136). E. faecalis uses a peptide signaling system to control plasmid conjugation (reviewed in reference 137). This signaling system triggers conjugation when a sufficient quorum of plasmid-free recipient cells is detected. In this case, albumin sequesters a peptide inhibitor that normally prevents conjugation in the absence of recipient cells (136). Growth in serum increased conjugation, presumably because albumin-dependent sequestration of the inhibitor caused conjugation to go unchecked (136). Results of these two studies suggest the outcome of QS-mediated species interactions might be very different in a host environment from that observed under standard laboratory growth conditions, an idea that requires further study in infections *in vivo*. A key challenge moving into polymicrobial infection models is to develop systems such as these that mimic the host environment in a context where variables, such as key nutrients and host-supplied factors, can be controlled or removed. In this way, the conditions and types of infections that drive cell-cell interactions can be delineated.

## CONCLUSIONS

There is now a wealth of knowledge of how bacterial populations use QS systems to communicate and coordinate diverse behaviors. In the past decade, this knowledge has served as a foundation to build approaches for studying QS in polymicrobial communities. This emerging field of investigation is relevant to our understanding of how QS contributes to the success of bacteria in diverse environments—from polymicrobial infections to natural communities—and how these systems might be manipulated to encourage specific outcomes, such as altering community dynamics of microbiomes or ecologically important soil communities. Advances in this field have relied on laboratory and *in vivo* models of nonclonal bacterial populations to model natural communities that can be prohibitively complex to study directly. By studying polymicrobial model systems, we have learned that QS is important for cooperation and for competition among and between species. These models have also been useful to test predictions about the evolution of QS and social behavior. We anticipate existing models will continue to provide new insights into QS and sociality, either as they are or when adapted for new purposes or increased complexity. We also look forward to results of studies with newly developed models (e.g., the three-dimensional protein-based picoliter-scale microcavities [termed bacterial “lobster traps” in reference 138], *in vitro* wound models [126], and alginate bead aggregates [139]) and their application to designing studies of more natural ecosystems and infections. Key questions include understanding how QS drives polymicrobial interactions across different host and nonhost environments and how these interactions drive the evolution of QS and ultimately shape the structure and behavior of these communities.
