# Erratum for Abisado et al., “Bacterial Quorum Sensing and Microbial Community Interactions”

**DOI:** 10.1128/mBio.01749-18

**Published:** 2018-10-02

**Authors:** Rhea G. Abisado, Saida Benomar, Jennifer R. Klaus, Ajai A. Dandekar, Josephine R. Chandler

**Affiliations:** a Department of Molecular Biosciences, University of Kansas, Lawrence, Kansas, USA; b Department of Microbiology, University of Washington, Seattle, Washington, USA

## ERRATUM

Volume 9, issue 3, e02331-17, 2018, https://doi.org/10.1128/mBio.02331-17. In Table 1 (Table 1 caption, Models for studying QS in cooperative and competitive microbial interactions), the Bacillus subtilis QS function indicated should be “surfactin” and not “rhamnolipid” as indicated. We thank Akos T. Kovacs (@EvolvedBiofilm) for notifying us of this error.

